# Allergen Immunotherapy in Asthma

**DOI:** 10.3390/pathogens10111406

**Published:** 2021-10-29

**Authors:** Kazuyuki Nakagome, Makoto Nagata

**Affiliations:** 1 Department of Respiratory Medicine, Saitama Medical University, Saitama 350-0495, Japan; favre4mn@saitama-med.ac.jp; 2 Allergy Center, Saitama Medical University, Saitama 350-0495, Japan

**Keywords:** allergen immunotherapy, bronchial asthma, subcutaneous immunotherapy, sublingual immunotherapy

## Abstract

Allergen immunotherapy (AIT) is a specific treatment involving the administration of relevant allergens to allergic patients, with subtypes including subcutaneous immunotherapy (SCIT) and sublingual immunotherapy (SLIT). In asthma, AIT using the house dust mite (HDM) alleviates clinical symptoms and decreases airway hyper responsiveness and medication dose. In addition, AIT can improve the natural course of asthma. For example, the effects of AIT can be preserved for at least a few years, even after ending treatment. AIT may increase the remission rate of asthma in children and suppress sensitization to new allergens. If AIT is introduced in pollinosis, AIT may prevent the development of asthma. Moreover, AIT can control other allergic diseases complicated by asthma, such as allergic rhinitis, which also improves the control of asthma. The indication of HDM-SCIT for asthma is mild-to-moderate HDM-sensitized allergic asthma in a patient with normal respiratory function. To date, HDM-SLIT is applicable in Japan for allergic rhinitis, not for asthma. However, the effect of SLIT on asthma has been confirmed internationally, and SLIT is available for asthma in Japan if allergic rhinitis is present as a complication.

## 1. Introduction

Allergen immunotherapy (AIT) is a treatment involving the administration of increasing doses of clinically relevant allergens to patients who have allergic disease [1]. The effect of AIT has been clinically confirmed in the cases of allergic asthma, allergic rhinitis, and hymenoptera hypersensitivity [1,2]. AIT shows subtypes such as subcutaneous immunotherapy (SCIT) and sublingual immunotherapy (SLIT). Allergens are subcutaneously injected in SCIT, or placed sublingually then swallowed in SLIT.

Generally, bronchial asthma is now a well-controlled disease due to advances in pharmacological therapies such as inhaled corticosteroid (ICS). However, ICS treatment does not improve the natural course of bronchial asthma and is, therefore, positioned as a symptomatic treatment [3,4]. For example, we reported that among 11 asthmatics who were well-controlled (without symptoms or exacerbation) on low-dose ICS, 10 showed increased airway hyper responsiveness (AHR) (91%) and four experienced clinical relapse of asthma (36%) within 1 year following ICS cessation [4]. In addition, ICS is ineffective for allergic rhinitis, which is often encountered as a complication in asthmatics. On the other hand, AIT may induce remission immunologically [1]. Furthermore, AIT is effective for other allergic conditions present concomitantly in allergic individuals.

AIT differs from pharmacotherapy in that the therapy can improve the natural course of asthma and allergies. Here, we discuss the efficacy of AIT and its role in treating asthma.

## 2. Effect of Subcutaneous Immunotherapy (SCIT) on Bronchial Asthma

Meta-analyses have confirmed that SCIT alleviates clinical symptoms of asthma and decreases AHR and medication dose [5,6], although some potential publication bias has been suggested. The standardized mean difference for asthma symptom scores by SCIT was −0.59 (95% confidence interval (CI) −0.83 to −0.35), compared to −0.53 (95%CI −0.80 to −0.27) for asthma medication scores and −0.61 (95%CI −0.79 to −0.43) for AHR [5].

**Figure 1:**
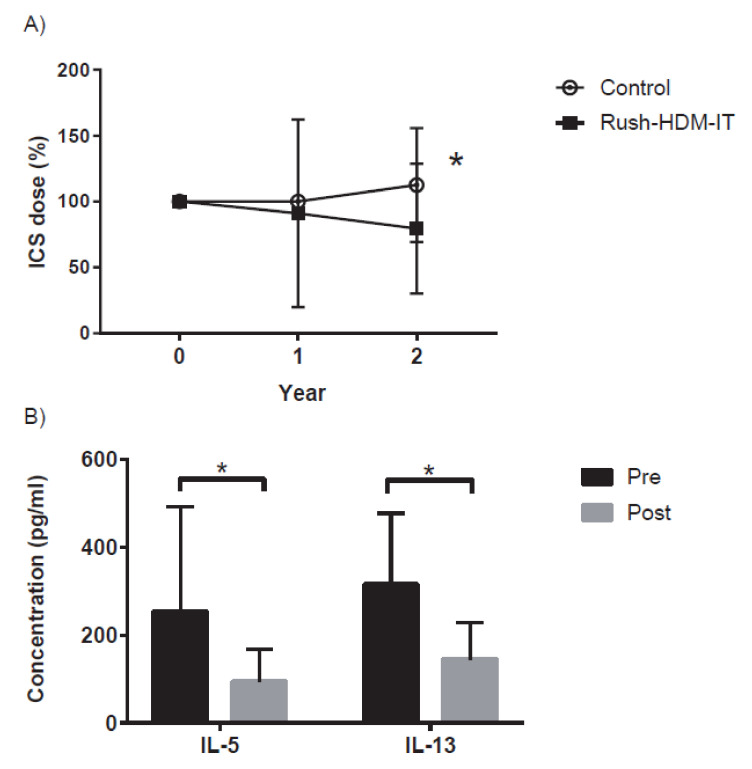
Addition of house dust mite subcutaneous immunotherapy (HDM-SCIT), introduced by the rush method, to guideline treatment provides clinical improvement in asthma with the inhibition of HDM-specific Th2-mediated systemic immune responses [8] (**A**) Changes in the dose of inhaled corticosteroid (ICS) after rush-HDM-IT and subsequent maintenance SCIT. The ICS dose before the induction of rush-IT or at a similar time point (Year 0) was used as a control (100%). * *p* < 0.05 when compared with Rush-HDM-IT. (**B**) *Dermatophagoides farinae*-induced IL-5 and IL-13 production from peripheral blood mononuclear cells (PBMCs) before and after rush-HDM-IT. * *p* < 0.05 when compared with cytokine production before rush-HDM-IT (Pre). ("Clinical evaluation of rush immunotherapy using house dust mite allergen in Japanese asthmatics" © Uchida T, et al. 2021 (Licensed under CC BY 4.0.); https://apallergy.org/DOIx.php?id=10.5415/apallergy.2021.11.e32, accessed on 30 August 2021).

The effect of adding SCIT using the house dust mite (HDM) (HDM-SCIT) to the standard treatment has also been confirmed [7]. The addition of HDM-SCIT reduced the use of inhalational β2-agonists and increased peak flow in mild-to-moderate HDM-sensitized asthma. Recently we reported that adding HDM-SCIT, introduced using rush methods, to the guideline treatment decreased the ICS dose and improved the treatment step of bronchial asthma with the inhibition of HDM-induced interleukin (IL)-5 and IL-13 production from peripheral blood mononuclear cells (PBMCs) (Figure 1) [8]. In childhood asthma, adding HDM-SCIT also decreased the ICS dose and increased peak flow [9]. Furthermore, SCIT using a modified HDM extract, which includes a depigmentation step and subsequent polymerization with glutaraldehyde and adsorption onto aluminum hydroxide, improved asthma symptoms and results from the allergen-specific bronchial provocation test [10,11,12]. Collectively, HDM-SCIT has additional effects even after performing guideline treatment. AIT also acts to suppress sensitization to new allergens, maintain effects for at least a few years even after treatment is discontinued, and control other allergic diseases such as allergic rhinitis, which is often seen as a complication in asthma. Taken together, AIT is considered to have clinical implications differing from those of pharmacotherapy as represented by ICS.

The United States adult asthma management guidelines (Expert Panel Report 3) and The 2020 Focused Updates to the Asthma Management Guidelines state that SCIT should be considered for allergic asthma in steps 2–4 (approximately equivalent to mild-to-moderate persistent asthma) of the six treatment steps [13,14]. The European Academy of Allergy and Clinical Immunology (EAACI) guidelines recommend HDM-SCIT as an add-on to regular asthma therapy for adults with controlled or partially controlled HDM-sensitized-allergic asthma [15].

## 3. Effect of Allergen Immunotherapy (AIT) on Asthma with Allergic Rhinitis

AIT is already the standard treatment for allergic rhinitis. Asthmatic patients are more likely to have allergic rhinitis [16]. In allergic rhinitis patients, nasal allergen challenge induces infiltration of eosinophils into the lower respiratory tract, smooth muscle contraction and AHR [17]. In contrast, in asthmatics without rhinitis, direct bronchial allergen challenge induces nasal eosinophilic inflammation [18]. The idea of “one airway, one disease”, that airway allergic inflammation can be deteriorated by nasal allergic inflammation and vice versa, is well known [19].

The treatment of allergic rhinitis alleviates the symptoms of asthma and decreases AHR and exacerbation in asthmatics complicated with allergic rhinitis [20]. We have reported that patients with uncontrolled asthma are aware that symptoms of asthma deteriorate when symptoms of rhinitis deteriorate, and tend to ameliorate after treatment for rhinitis [21]. Consequently, treatment of rhinitis is crucial for managing asthma if complicated by rhinitis, and AIT represents a reasonable measure to manage rhinitis as well as asthma.

## 4. Introduction of House Dust Mite (HDM)-SCIT for Asthma

The indication of HDM-SCIT for asthma is mild-to-moderate-allergic asthma with normal respiratory function (percentage predicted forced expiratory volume in 1 second (%FEV_1_) ≥ 70%). SCIT is initiated in the clinically stable period. It is important to confirm by allergen testing (skin test and specific immunoglobulin (Ig) E antibody (Ab) test) and/or clinical history that the individual is sensitized to HDM allergen and that this represents a contributing factor to symptom onset and worsening. Generally, a comfortable effect is expected in a patient who is mono-sensitized to HDM. We reported that the clinical effect of SCIT is low in patients with a disease period more than 10 years or FEV_1_ < 70% [22]. Collectively, AIT is assumed to be more effective if applied in the early stage of allergic asthma, before the development of airway remodeling. In addition, as described above, a simultaneous effect on allergic rhinitis is expected in patients with asthma complicated by rhinitis.

The effect will be low in patients who are sensitized to pet allergens and keep pets, or in those sensitized to other perennial allergens such as fungi. The indications for treatment should, therefore, be judged carefully. As the effect of ICS is lower in patients with cigarette smoking, the effect of AIT is presumably not maximally exerted in smoking patients.

The initial concentration of allergen injection is the threshold of the intradermal test or one-tenth of that. The allergen should be injected subcutaneously in the forearm or upper arm. Double-checking the concentration and amount of allergen to be administered is important, especially when increasing the concentration or changing the lot. In the conventional schedule of SCIT, the amount of allergen usually increases once or twice a week by 50–100%. During the initial buildup phase, the patient should be monitored for 30 min after injection, and the diameter of the immediate skin reaction (redness and swelling) at the injection site is measured. The final dose as the increase achieved without side effects after a certain number of injections is used as the maintenance dose. After reaching the maintenance dose, allergen injection is repeated once every two weeks several times, then when the diameter of the skin-swelling decreases, once every 4 weeks for 3 years or more.

Conventional SCIT schedule requires weekly clinic visits for several months in the build-up phase. Adhering to the schedule can represent a significant burden in initiating SCIT in patients with allergic disorders [23,24,25]. To address this problem, rush SCIT schedules have been introduced to minimize the treatment schedule, particularly the buildup phase. In rush SCIT, the maintenance dose is easily reached by performing injections several times daily intensively within several days [23,24,25], and immediate effects can be expected.

## 5. Safety of HDM-SCIT

Generally, SCIT has a risk of one systemic side effect per 500–1000 injections (0.1–0.2%), and one fatal side effect per 1–2.5 million injections. An American Academy of Allergy, Asthma and Immunology/American College of Allergy, Asthma and Immunology surveillance study demonstrated one systemic reaction in about 1,000 injections (0.1%), one case of severe anaphylaxis (Grade 4; anaphylactic shock) in about 1 million injections, and one death in about 23 million injections [26].

A meta-analysis assessing HDM-SCIT in asthma reported that the incidence of systemic response per patient is 5–7% [27]. Generally, systemic reactions to HDM-SCIT, including anaphylaxis, have been observed in 0.2–0.4% of injections and 2–10% of patients. Ohashi et al. reported that the frequency of serious or severe systemic reactions by HDM-SCIT requiring special treatment was 0.12% of injections [28]. Although few data have been accumulated on systemic reactions to SCIT using standardized HDM at present, the possibility that side effects appear more easily than with other allergens has not been excluded.

In SCIT, side effects often occur within 30 min after administration, but can be seen even after that. Additionally, side effects often occur during build-up periods, but can be seen even in the maintenance period. Oral administration of histamine H1 receptor antagonists before each injection may reduce the risk of serious side reactions.

## 6. Mechanisms and Biomarkers of AIT

AIT induces the generation of allergen-specific IgG or IgG4 and IgA Abs in serum [29,30,31,32]. Several studies have suggested a suppressive effect of IgG or IgG4 in IgE-dependent cell activation. IgG or IgG4 competes with IgE, and thus inhibits the formation of allergen-IgE complexes [33]. AIT can therefore suppress the activity of basophils and mast cells such as histamine release by inhibiting the cross-linking of high-affinity IgE receptors (FcεRI). In addition, AIT can suppress IgE-facilitated allergen presentation of B cells to T cells by inhibiting the binding of allergen-IgE complexes to low-affinity receptors (FcγRIIb) [34].

AIT suppresses local numbers of Th2 cells or production of Th2 cytokines, such as IL-4 and IL-5 [35,36,37]. We confirmed that AIT suppresses allergen-induced production of IL-5 and IL-13 from PBMCs obtained from patients with HDM-sensitized-allergic asthma (Figure 1) [8]. AIT also inhibits the allergen-induced thymus and activation-regulated chemokine production from PBMCs in allergic asthma [1]. As a result, AIT can decrease the accumulation of Th2 cells in the airways. Further, AIT increases regulatory T cells (Tregs) including natural Tregs associated with the expression of transcription factor forkhead box P3 (FOXP3) and inducible Tregs associated with the production of IL-10, transforming growth factor (TGF)-β, and IL-35 [29,38,39,40]. AIT induces local FOXP3^+^ T cells [38,39], IL-10^+^ [30,40] and TGF-β^+^ T cells [29]. However, the contribution of Tregs in AIT to the suppression of Th2-type immune responses may be regulated by multiple factors, including the type of allergen, time of assessment and method of evaluation. Furthermore, recent studies have focused on the roles of regulatory B cells associated with the induction of IL-10 [41,42]. In addition, AIT enhances allergen challenge-induced cutaneous IL-12 mRNA expression [43]. Therefore, AIT inhibits T cell-mediated allergic inflammation by suppressing Th2 cells and by inducing Tregs or Th1 cells. Moreover, AIT reportedly decreases the number of type-2 innate lymphoid cells as sources of Th2 cytokines in blood [44], although this remains controversial [45].

The exploration of predictive biomarkers for the efficacy of AIT is of crucial importance. The ratio of specific IgE to total IgE in serum at baseline, changes in the concentration of allergen-specific IgG4 in serum, changes in IgE-FAB, and changes in expression of basophil activation markers are important candidates for predictive biomarkers [46,47].

Allergen-specific IgE concentrations increase transiently during AIT, then decrease gradually over several years [31,48,49]. However, no relationship has been identified between changes in allergen-specific IgE concentrations and those in clinical responses [32,40]. On the other hand, several studies have reported that the ratio of specific IgE to total IgE in serum before treatment is associated with the clinical efficacy of AIT [50,51]. 

Allergen-specific IgG or IgG4 increase during AIT [52,53,54]. We reported that the induction of HDM-specific IgG4 is correlated with the amelioration of AHR in asthma [1].

IgG-mediated IgE-inhibition could be evaluated by the IgE-FAB system [47,55]. The IgE-FAB system assesses the capacity of serum including IgG to suppress FcεRII-mediated binding of allergen-IgE to B cells [31,47]. Another assay is enzyme-linked immunosorbent-facilitated antigen-binding (ELIFAB) [47,56]. The change of IgE-FAB and ELIFAB correlates more closely with the clinical efficacy of AIT as compared with that of serum IgG or IgG4 [31,47,56], as IgE-FAB or ELIFAB indicates the function of Ab binding.

## 7. Effects of AIT on Natural Course of Allergic Disease

Importantly, AIT can modify the natural course of allergic diseases, unlike other pharmacotherapies. Moreover, AIT is still effective for at least a few years even after the treatment discontinuation. For example, 3–4 years of AIT for hay fever results in symptom-relieving effects for 3 years after treatment discontinuation [57]. Furthermore, 3 years of AIT for rhinoconjunctivitis ameliorates symptoms and inhibits allergen challenge reaction in the conjunctiva for 7 years after the end of treatment [58]. Furthermore, in childhood asthma patients with allergic rhinitis, 5 years of AIT increases the frequency of the remission of asthma, and remission is preserved for 5 years after the end of treatment [59].

AIT has the clinical effect of suppressing sensitization to further allergens, although patients with allergic asthma tend to be sensitized with new allergens. Marogna et al. reported from a 15-year observational study that allergic patients treated with pharmacological therapies alone were all sensitized with new allergens after 15 years (100%). In contrast, 3–5 years of AIT decreased the rate of new allergen sensitization to 12–21% [60].

AIT also has an effect on preventing asthma onset in children with pollinosis. In a 3-year observational study of children with pollinosis, AIT suppressed the development of asthma [61]. Furthermore, this prevention was preserved even 7 years after the end of AIT. That study demonstrates that AIT can decrease the risk of asthma development in patients with allergic rhinitis.

Some negative reports have described the inhibitory effects of AIT on allergen sensitization and asthma onset, but need to be carefully scrutinized. For example, a meta-analysis by Di Bona et al. did not demonstrate evidence supporting effects to prevent sensitization to new allergens in pediatric patients [62]. They reported that the level of evidence was low and the risk of bias was high, and the preventive effect was recognized in small-scale studies and studies with a short follow-up period. As for the effect in preventing new allergen sensitization among children, they described six papers reporting that AIT was more effective, three papers reporting that the effect was unchanged, and two papers [63,64] reporting that pharmacotherapy was more effective [62]. However, one of the two papers claiming that pharmacotherapy was effectively had an incorrect conclusion (the original paper reported that AIT was more effective) [63], and the other paper was unclear about the effects of AIT (whether a sufficient therapeutic dose was administered) [64]. Analysis of sensitization-preventing effects in this meta-analysis was thus considered inappropriate.

## 8. Effect of Sublingual Immunotherapy (SLIT) on Bronchial Asthma

As there is a risk of systemic reactions in SCIT, SLIT was developed as a safer procedure. In the 1990s, SLIT using HDM (HDM-SLIT) was reported to alleviate symptoms and decrease AHR in HDM-sensitized asthma [65]. Furthermore, in asthmatics with rhinitis by pollinosis, SLIT alleviates asthma symptoms, improves respiratory function and reduces bronchodilator use. Moreover, Marogna et al. examined the effects of SLIT as compared with that of ICS in mild asthmatics complicated by rhinitis due to pollinosis [66]. Patients were randomized to either receive ICS or SLIT for 5 years. Although both ICS and SLIT improved asthma symptoms, patients on SLIT showed a greater degree of improvement as compared to ICS. Furthermore, SLIT suppressed both rhinitis symptoms and nasal eosinophilia.

In terms of efficacy, SLIT may be inferior to conventional SCIT [67]. However, SLIT is widely used in clinical practice worldwide because of its convenience and safety.

The effect of the HDM-SLIT tablet by the Danish ALK on bronchial asthma was established in large-scale clinical studies [68,69]. This tablet (6 standardized quality (SQ)) decreases the requirements for ICS (SLIT 42%, placebo 15%) and increases the rate of ICS discontinuation (SLIT 34%, placebo 21%) [68]. Furthermore, 6SQ or 12SQ for this tablet decreases moderate-to-severe exacerbations of asthma induced by ICS reduction [69]. With reference to such evidence, the Global Initiative for Asthma reports that HDM-SLIT should be considered in adult HDM-sensitized patients with allergic rhinitis provided %FEV_1_ is more than 70% [70].

One important problem of SLIT that should be addressed is adherence. More than 3 years of treatment are needed to improve the natural course of allergic disease. However, Sena et al. reported that sales from SLIT prescriptions decreased from 100% to 44% in the first year, to 28% in the second year, and to 13% in the third year, suggesting that less than 20% were continuing after 3 years [71]. Moreover, differences were seen between regions with full reimbursement and those with no reimbursement in the second and third years [71]. Therefore, we should investigate the causes and urgently address this problem. However, differences in adherence to SLIT may exist in Japan, as described below.

## 9. AIT in Japan

In SCIT, we could not use the standardized HDM allergen until 2015. Before 2015, we used house dust, which is from ordinary Japanese houses, as an alternative. Although the important component of house dust was mites, product quality issues were seen, necessitating improvements in the efficacy and safety by allergen standardization. A standardized HDM allergen was approved in 2015 in our country, and is still in use for the asthma treatment.

In SLIT, two types of tablets of HDM-SLIT were approved for allergic rhinitis in 2015 in our country, but not for asthma. One tablet (MITICURE^®^, Torii Pharmaceutical Co., Ltd, Tokyo, Japan) is the same as that by ALK, for which an effect on asthma control and exacerbation has been demonstrated as described above (10,000 Japanese allergy units (JAU), the maintenance dose, is equivalent to 6SQ in Europe).

Recently, the effects of MITICURE^®^ have also been reported in our country. The addition of MITICURE^®^ improves asthma symptoms and respiratory function, and decreases fractional exhaled nitric oxide and airway wall thickening on chest CT in HDM-sensitized allergic asthma with rhinitis [72]. HDM-SLIT therefore inhibits both airway inflammation and remodeling. In addition, in patients used short-acting β2-agonists during the observation period, MITICURE® suppressed asthma exacerbations induced by ICS reduction [73], consistent with the study in Europe [69].

Pollinosis caused by Japanese cedar pollen (JCP) is an important seasonal rhinitis in our country. The prevalence of pollinosis due to JCP was 26.5% in 2008 [2] and has increased by about 10% in 10 years. Like other pollens, JCP has been reported to worsen the control of asthma [74]. 

For JCP-induced asthma exacerbation, we observed that JCP-SLIT abolished the exacerbation of asthma in the JCP-scattering season [75], supporting the preventive effects of SLIT on the exacerbation of asthma. HDM- and JCP-SLIT are, therefore, useful for managing bronchial asthma with rhinitis.

As for adherence to SLIT, the treatment continuation rate can be high in Japan. Yuta et al. reported that 83% of patients reveal good adherence of JCP-SLIT as calculated from prescriptions for 2 years [76]. Although the reason for the discrepancy is unknown, the symptoms of JCP-induced allergic rhinitis may be severe and not sufficiently relieved by pharmacological treatment only, so patients may have wanted to receive JCP-SLIT. We recently performed a prospective assessment of predictors for adherence to JCP-SLIT and found that patients younger than 40.5 years old demonstrated poor adherence to JCP-SLIT [77].

## 10. Conclusions

In HDM-sensitized mild-to-moderate asthma, HDM-SCIT alleviates clinical symptoms of asthma and decreases medication dose. In addition, HDM- or JCP-SLIT can reduce asthma exacerbations and medication dose. Pharmacological therapies centered on ICS do not improve the natural course of asthma and allergy, whereas AIT can induce immunological remission, including type 2 immune responses. Therefore, AIT needs to be more widely used in asthma treatment as a comprehensive management for allergic diseases and modification of their natural course.
